# The “Bankart knee”: high-grade impression fractures of the posterolateral tibial plateau lead to increased translational and anterolateral rotational instability of the ACL-deficient knee

**DOI:** 10.1007/s00167-023-07432-w

**Published:** 2023-05-08

**Authors:** Danko Dan Milinkovic, Christoph Kittl, Elmar Herbst, Christian Fink, Friedrich Greis, Michael J. Raschke, Robert Śmigielski, Mirco Herbort

**Affiliations:** 1 Center for Musculoskeletal Surgery, Charité-University Medicine Berlin, Charitéplatz 1, Luisenstrasse 64, 10117 Berlin, Germany; 2 Department of Trauma-, Hand- and Reconstructive Surgery, Westfaelian–Wilhelms University of Muenster, Munster, Germany; 3 Gelenkpunkt Sportsclinic, Innsbruck, Austria; 4 Research Unit for Orthopaedic Sports Medicine and Injury Prevention, Institute for Sports Medicine, Alpine Medicine and Health Tourism, Private University for Health Sciences, Medical Informatics and Technology, Hall in Tirol, Austria; 5 Clinic for General Orthopedic and Tumor Orthopedic Surgery, Westfaelian–Wilhelms University of Muenster, Munster, Germany; 6 Life Institute, Warsaw, Poland; 7 OCM Orthopedic Surgery Munich Clinic, Munich, Germany

**Keywords:** Posterolateral tibial plateau fractures, Biomechanics, ACL deficiency, Injuries concomitant with ACL rupture

## Abstract

**Purpose:**

The aim of this biomechanical cadaver study was to evaluate the effects of high-grade posterolateral tibia plateau fractures on the kinematics of anterior cruciate ligament (ACL)-deficient joints; it was hypothesized that, owing to the loss of the integrity of the osseous support of the posterior horn of the lateral meniscus (PHLM), these fractures would influence the biomechanical function of the lateral meniscus (LM) and consequently lead to an increase in anterior translational and anterolateral rotational (ALR) instability.

**Methods:**

Eight fresh-frozen cadaveric knees were tested using a six-degree-of-freedom robotic setup (KR 125, KUKA Robotics, Germany) with an attached optical tracking system (Optotrack Certus Motion Capture, Northern Digital, Canada). After the passive path from 0 to 90° was established, a simulated Lachman test and pivot-shift test as well as external rotation (ER) and internal rotation (IR) were applied at 0°, 30°, 60° and 90° of flexion under constant 200 N axial loading. All of the parameters were initially tested in the intact and ACL-deficient states, followed by two different types of posterolateral impression fractures. The dislocation height was 10 mm, and the width was 15 mm in both groups. The intraarticular depth of the fracture corresponded to half of the width of the posterior horn of the lateral meniscus in the first group (Bankart 1) and 100% of the meniscus width in the second group (Bankart 2).

**Results:**

There was a significant decrease in knee stability after both types of posterolateral tibial plateau fractures in the ACL-deficient specimens, with increased anterior translation in the simulated Lachman test at 0° and 30° of knee flexion (*p* = 0.012). The same effect was seen with regard to the simulated pivot-shift test and IR of the tibia (*p* = 0.0002). In the ER and posterior drawer tests, ACL deficiency and concomitant fractures did not influence knee kinematics (n.s.).

**Conclusion:**

This study demonstrates that high-grade impression fractures of the posterolateral aspect of the tibial plateau increase the instability of ACL-deficient knees and result in an increase in translational and anterolateral rotational instability.

**Supplementary Information:**

The online version contains supplementary material available at 10.1007/s00167-023-07432-w.

## Introduction

Historically, the first reports of osteochondral lesions associated with ACL tears can be found in several publications dating from the early 1990s [20, 30]. The occult fractures described in these studies were primarily of the bone bruise type, sometimes referred to as nondisplaced fractures. Their typical localization in the anterior to middle segment of the lateral femoral condyle (LFC) and the middle to posterior segment of the lateral tibial plateau (LTP) reflects the mechanism of injury to the ACL, in which the lateral tibial plateau subluxates anteriorly, resulting in a violent collision between the anterolateral aspect of the LFC and the posterolateral area of the tibial plateau (PLTP) [7, 8, 12, 22, 31, 40].

However, an impression fracture of the tibial plateau, seen as a depression of the articular surface with a breach of the cortical bone, is considered to be an injury of much greater severity than isolated bone contusions [3–6, 13, 37]. To our knowledge, there are no studies in the current literature investigating the biomechanical influence of these fractures on the kinematics of ACL-deficient joints. However, several biomechanical studies have already shown the important role of the posterior horn and roots of the LM on knee kinematics, especially in terms of anterior tibial translational (ATT) and anterolateral rotational (ALR) stability [14, 24, 34, 37]. It has been reported that the posterior horn of the lateral meniscus (PHLM) acts as a wedge to stop the LFC during ATT and internal rotation (IR) of the tibia [14, 17, 24, 34]. The intact tibial plateau provides osseous support for the PHLM and is therefore essential for maintaining its stability and biomechanical function [24, 27, 34]. A similar effect was demonstrated in the shoulder joint, where, in cases of a bony Bankart lesion characterized by an impression of the glenoid fossa, the consequential loss of osseous support for the labrum causes its biomechanical function to change [21, 30]. By analogy between the biomechanical influences of a bony Bankart lesion and those of the knee fractures in this study, this type of knee injury is referred to as “the Bankart knee”.

This study assessed the influence of PLTP impression fractures on the knee kinematics in an ACL-deficient state. The hypothesis was that the fractures causing high-grade depression of the tibial articular surface would affect the biomechanical function of the lateral meniscus, primarily due to the loss of bony support of the PHLM, resulting in an increase in ATT and ALR instability of the ACL-deficient joint.

## Materials and methods

**Fig. 1:**
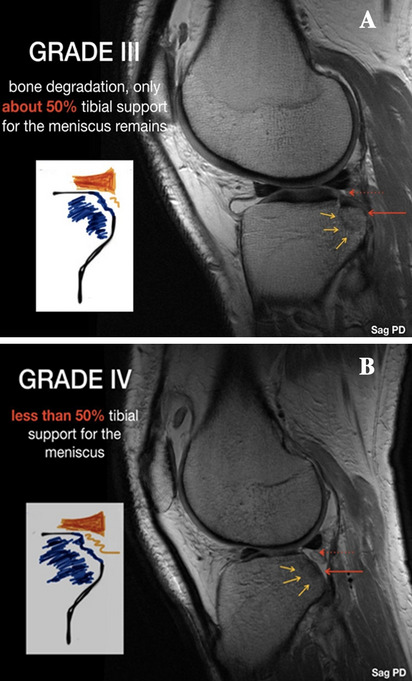
Typical impression fracture of the posterolateral zone of the tibial plateau according to Smigielski classification, seen in the sagittal plane MRI sequence of the right knee; **A** Grade III fracture with around 50% of remaining osseous support.; **B** Grade IV fracture with less than 50% of remaining osseous support

Eight fresh-frozen unpaired cadaveric knees were used for the current study (four female/four male; mean 77.5 ± 7 years of age). All specimens were obtained from a local tissue bank and tested with the permission of the local district law (ID No. 2128-2) according to the “Gesetz ueber das Leichen-, Bestattungs- und Friedhofswesen (Bestattungsgesetz) des Landes Schleswig–Holstein vom 04.02.2005, Abschnitt II, § 9 (Leichenoeffnung, anatomisch)”. In this case, it is permissible to dissect the bodies of donors for scientific and/or educational purposes without seeking further approval. Therefore, no approval of the ethics committee was needed. In the specimen inclusion process, only knees that showed no signs of previous trauma, surgical procedures and/or high-grade ligamentous instability were selected from the local tissue bank. Knees with osteopenia and poor bone quality were excluded following a radiographic investigation in two planes. All of the tested specimens were stored in a polyethylene bag at − 20 °C and left to thaw at room temperature for 24 h prior to testing (Fig. 1). The knees were kept moist during testing to prevent desiccation [38].

### Specimen preparation

**Fig. 2:**
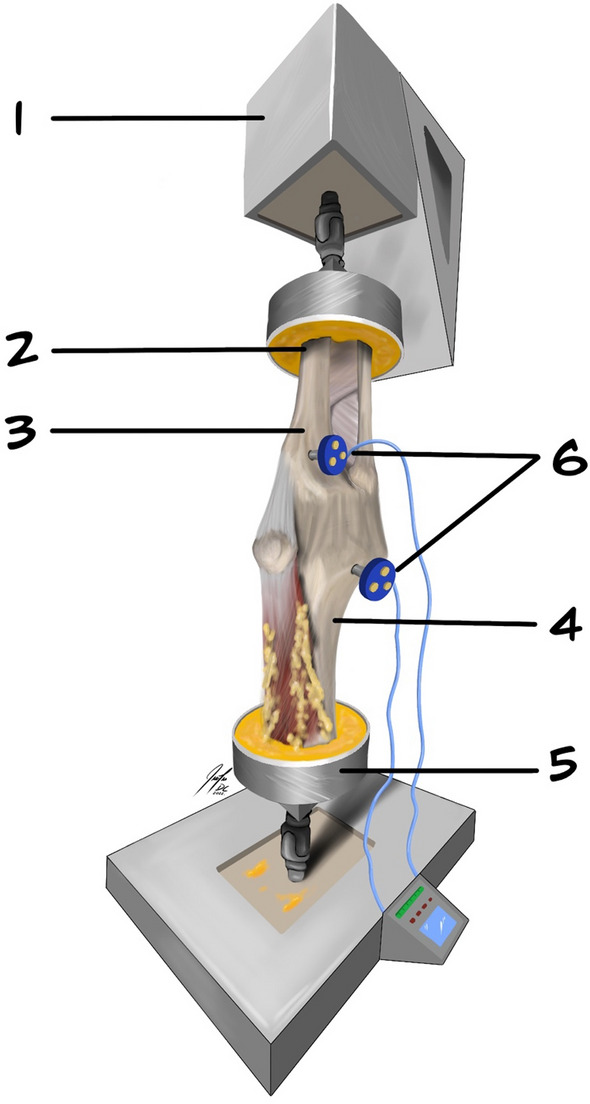
Biomechanical setup with fixed left cadaveric knee, with: (1) movable component- the robotic arm; (2) aluminium tube; (3) tibia fixed in an upside-down orientation; (4) femur fixed in an upside-down orientation; (5) static component of the robot; (6) two optical sensors fixed to the shaft of tibia and femur with custom-made pins

In each of the tested specimens, the femur and tibia were cut approximately 20 cm from the joint line. The dissection was initiated through a midline incision through the cutaneous and subcutaneous tissue. All of the soft tissue structures up to approximately 10 cm from the joint line were removed, revealing the shaft of the tibia and femur. The remaining fascia, muscles and ligaments were left intact (Fig. 2). Special care was taken to leave a significant portion of the iliotibial tract (ITT), as well as the structures of the posterolateral corner of the knee, intact. The insertion of the posterior cruciate ligament (PCL) on the posterolateral margin of the tibia was identified, followed by a small horizontal incision (approx. 0.5 cm) through the posterolateral capsule just lateral to the tibial insertion of the PCL. This allowed for intraarticular measurement of the width of the PHLM using a standard small screw depth gauge. Once the testing was concluded, the measurements were reconfirmed using an electronic calliper (± 0.2 mm), and no measurement errors were found in any of the specimens. The head of the fibula was fixed at the proximal tibiofibular joint with a 4 mm cortical screw, and the remaining length of the bone was removed. The insertions of the LCL and biceps femoris muscle (BFM) on the fibular head were left intact. A modified patellar arthrotomy along the midline of the supero-inferior axis of the patella was performed using a fine oscillating saw. The arthrotomy was completed with a scalpel, and the split was extended into the patellar tendon. This approach allows direct visualization and palpation of the femoral insertions of the ACL and PCL, as well as access to and dissection of the ACL during testing. This longitudinal transpatellar approach was previously outlined as a reliable and convenient approach to the knee joint for in vitro studies, without any significant effect on the surrounding tissue and overall kinematics of the joint [29, 36, 38]. The bony approach to the intraarticular space was closed and fixed using two 4-mm cancellous screws. The tibia was then rigidly fixed in an aluminium tube (cylinder) using polymethyl methacrylate bone cement (PMMA). The tibia was centred in the cylinder so that its axis was aligned with the condylar eminence at the centre of the tibial plateau [23]. Additional 4-mm screws were added to prevent possible sliding out of the tibial shaft during testing (Fig. 2). The described preparation and fixation procedure have previously been published [23, 36, 38].

### Biomechanical setup

A six-degree-of-freedom (DOF) robot was used for biomechanical testing (6DOF; KR 125; KUKA Robotics, Augsburg, Germany). Additionally, a force/moment sensor (UFS; FTI Theta 1500–240; Schunk, Lauffen, Germany) was mounted on the robotic rig. The repeatability and accuracy of the KUKA robot system are 0.2 mm and 0.02° for the orientation and position of the end effector, respectively, whereas the maximum loading capacity is 2450 N [36, 38]. The UFS resolution was 0.25 N and 0.05 Nm for forces and torques, respectively. To track the kinematics of the knee movement through simulated stress tests, an optical tracking system with an accuracy of up to 0.1 mm and a resolution of 0.01 mm was used (Optotrak Certus Motion Capture, Northern Digital, Ontario, Canada). Two sensors were rigidly affixed to the tibial tuberosity and the shaft of the femur using custom-made pins (Fig. 2). The knee coordinate system was defined by six tibial (medial/lateral tibial shaft and medial/lateral/anterior/posterior tibial plateau) and four femoral landmarks (medial/lateral femoral shaft and medial/lateral femoral epicondyle). Each of the anatomic landmarks was digitized with a generic digitizing probe. This allowed 6-DOF kinematics to be computed in terms of rotations and translations. Visual 3D computer software (C-Motion Inc., Germantown, Maryland) was used for data processing. This software designs a digitized coordinate system of the tibia using the Cardan *x–y–z* axis model [36, 38]. The digitized landmarks were used to create static and moving knee models at each of the tested flexion angles. The difference in translation (mm) or rotation (degree) between the static and moving models at the end of each simulated laxity test in all degrees of flexion (0°, 30°, 60° and 90°) was recorded and compared [38]. The specimens were fixed in the robot in an upside-down orientation by attaching the aluminium tube with the fixed tibia to the movable component (arm) of the robot and fixing the femur in a tube placed in the stationary base of the machine (Fig. 2). The force vectors and moments were minimized to < 1 N and < 0.5 Nm, respectively. Prior to testing, each specimen was passively brought from full extension to 90° flexion in 1° increments. Throughout this passive motion, the robot was programmed to minimize forces acting upon the joint. The position of the knee at each flexion angle was recorded, and the passive path was repeated three times. Through the testing protocol, the specimens were placed under a constant 200 N axial compressive load. Once the passive path was established, the specimens could be moved automatically by the robotic arm to predetermined flexion angles and submitted to laxity tests while the remaining acting forces and torques remained at a minimum. The specimens were submitted to the following simulated knee laxity tests: The Lachman test (89 N), the posterior drawer test (89 N), internal rotation (IR; 4 Nm of rotational torque), and external rotation (ER; 4 Nm of rotational torque) were performed at 0°, 30°, 60° and 90° of knee flexion. The simulated pivot-shift test, corresponding to 4 Nm tibial internal rotation and 8 Nm valgus torque, was conducted at 0° and 30° of flexion. This method for robotic knee testing has been based on the overall principles of superposition as previously validated and published in studies by Guenther et al. [15, 16]. To differentiate the movement of the lateral tibial plateau in response to this dynamic multiplanar test, we distinguished between maximum anterolateral translation (ALT) of the midpoint of the lateral tibial plateau and anterolateral rotation (ALR) of the tibia, defined as the maximum IR occurring around the longitudinal axis of the tibia. The testing intervals were repeated three times for each of the 4 cutting states, i.e. the intact, ACL-deficient, 1st-degree depression fracture (Bankart 1) and 2nd-degree depression fracture (Bankart 2) states. The midpoint of the lateral tibial plateau was taken as ¼ of the maximum distance between the lateral and medial digitized points on the plateau, as previously published [38].

### Tissue cutting protocol and testing sequence

**Fig. 3:**
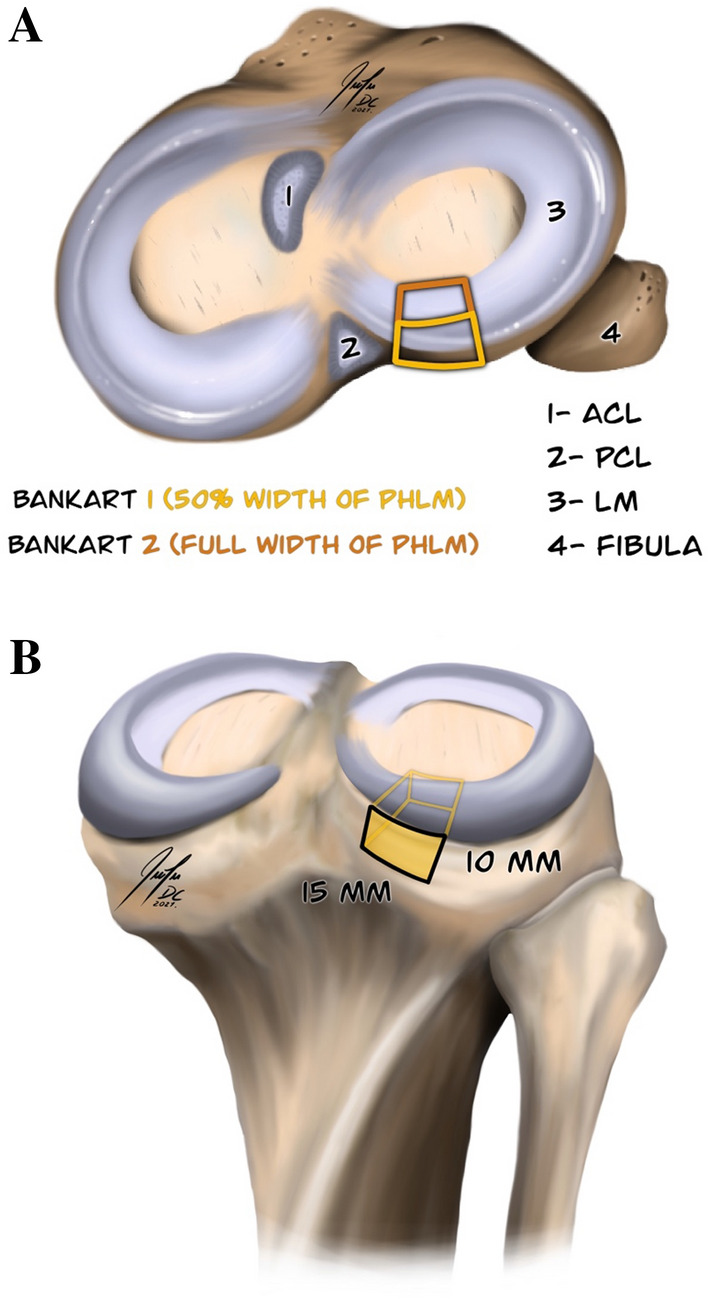
**A** Axial view of the tibial plateau with: (1) tibial insertion of the Anterior cruciate ligament; (2) tibial insertion of the Posterior cruciate ligament; (3) lateral meniscus; (4) fibula; and two degrees of the posterolateral impression fracture marked “Bankart 1” (yellow colour) and “Bankart 2” (orange colour). **B** Posterior view of the tibia showing standard proportions of the posterolateral impression fractures of the tibial plateau used in each of the tested specimens with: 15 mm in width and 10 mm in height

The specimens were initially tested in the intact state, followed by sequential cutting of the ACL and an additional 2 degrees of posterolateral depression fractures (marked Bankart 1 and 2) of the tibial plateau. The same cutting sequence was applied to all tested specimens. During cutting procedures, the specimens were kept mounted on the robot. Initial cutting of the ACL was performed through the preformed patellar split at 90° of knee flexion. The ACL was separated from its femoral insertion under direct visualization using a scalpel and scissors. Once the cut was made, the patella was fixed with two 4-mm screws. An initial fracture labelled Bankart 1 was made using a sharp 15-mm-wide chisel just lateral to the insertion of the PCL on the posterior edge of the tibial plateau. Standardized proportions for the plateau depression were applied to all tested specimens. The height of the depression was held constant at 10 mm, and the width was 15 mm starting from the point just lateral to the tibial insertion of the PCL (Fig. 3B). The intraarticular depth of the initial fracture, labelled Bankart 1, corresponded to half of the predetermined width of the PHLM, whereas the depth of the second depression fracture, labelled Bankart 2, corresponded to the full width of the PHLM (Figs. 1 and 3A).

### Statistical analysis

Before the statistical tests were performed, the data were checked for normality (using the Shapiro–Wilk normality test and by visual inspection using a QQ plot), and extreme outliers were removed (based on the boxplot method, i.e. values above Q3 + 3 × IQR or below Q1–3 × IQR).

Two-way repeated-measures ANOVA with post hoc Bonferroni corrections for multiple comparisons was performed to evaluate the effect of different cutting states over different knee flexion angles on tibial translation/rotation. The two independent factors were the four states (intact, ACL-deficient, Bankart 1 and 2) and the knee angle (0°, 30°, 60° and 90°) within the same specimen, while the dependent variable was the resulting translation/rotation. In the cases where the two-way repeated-measures ANOVA did not detect a statistically significant interaction effect of the cutting state and the flexion angle on translation or rotation of the tibia, one-way repeated-measures ANOVA was performed to evaluate the effect of the different flexion angles or different cutting states on the translation/rotation separately. One-tailed paired Student’s t tests were used to compare the mean translation/rotation between the fixed state/angle combinations. The significance level was set to 0.05. This analysis was performed using R (R Core Team, 2020) with additional packages (see Supplement file).

A post hoc power analysis was performed using G*Power according to the study by Faul et al. [11] using the results of the simulated pivot shift test at 0° and 30° of flexion in all specimens. Based on the data reported in that study, effect sizes of 0.5 and 0.8 were obtained at 0° and 30°, respectively. With these effect sizes and an alpha of 0.05, the power was 0.85 and 0.99, respectively.

## Results

### Lachman test: anterior tibial translation (89 N)

Regarding the simulated Lachman test, there was a statistically significant interaction between effect of state (ACL-deficient, Bankart 1, and Bankart 2) and angle on ATT (*p* = 0.012, eta2[g] = 0.04).

**Fig. 4:**
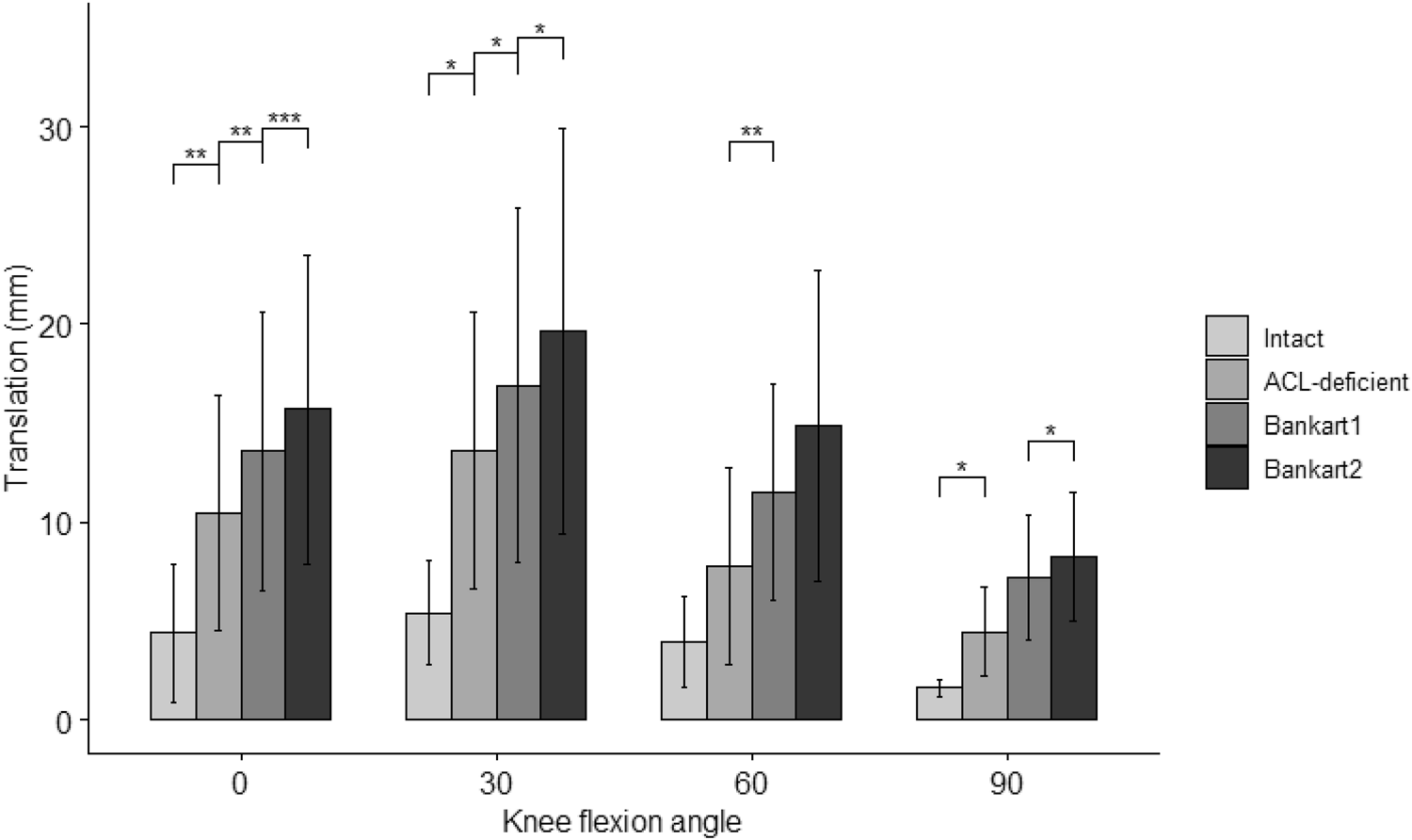
Anterior translation of the tibia in Intact, ACL-deficient, Bankart 1 and 2 States at 0–90° of knee flexion. Statistically significant differences compared to the previous states are indicated (*p < 0.05, **p < 0.01, ***p < 0.001). Error bars indicate the standard deviation

Pairwise comparisons showed that the mean ATT was statistically significantly different between all cutting states at angles of 0° and 30°, between the ACL-deficient and Bankart 1 states at 60°, between the intact and ACL-deficient states at 90°, and between the Bankart 1 and Bankart 2 states at 90° (Fig. 4 and Table 1).

### Posterior drawer test (89 N) and external rotation test (4 Nm of rotational torque)

Regarding posterior tibial translation, there was no statistically significant interaction between effect of state and angle on translation (n.s.). Furthermore, the effect of the cutting state was not statistically significant at any fixed flexion angle (n.s.).

Regarding ER, there was no statistically significant interaction between effect of cutting state and knee flexion angle on the ER of the tibia (n.s.). The effect of the cutting state was not statistically significant at any flexion angle, and pairwise tests did not show any difference between the mean rotation values for different states at any fixed angle (n.s.).

### Internal rotation (IR, 4 Nm of rotational torque)

**Fig. 5:**
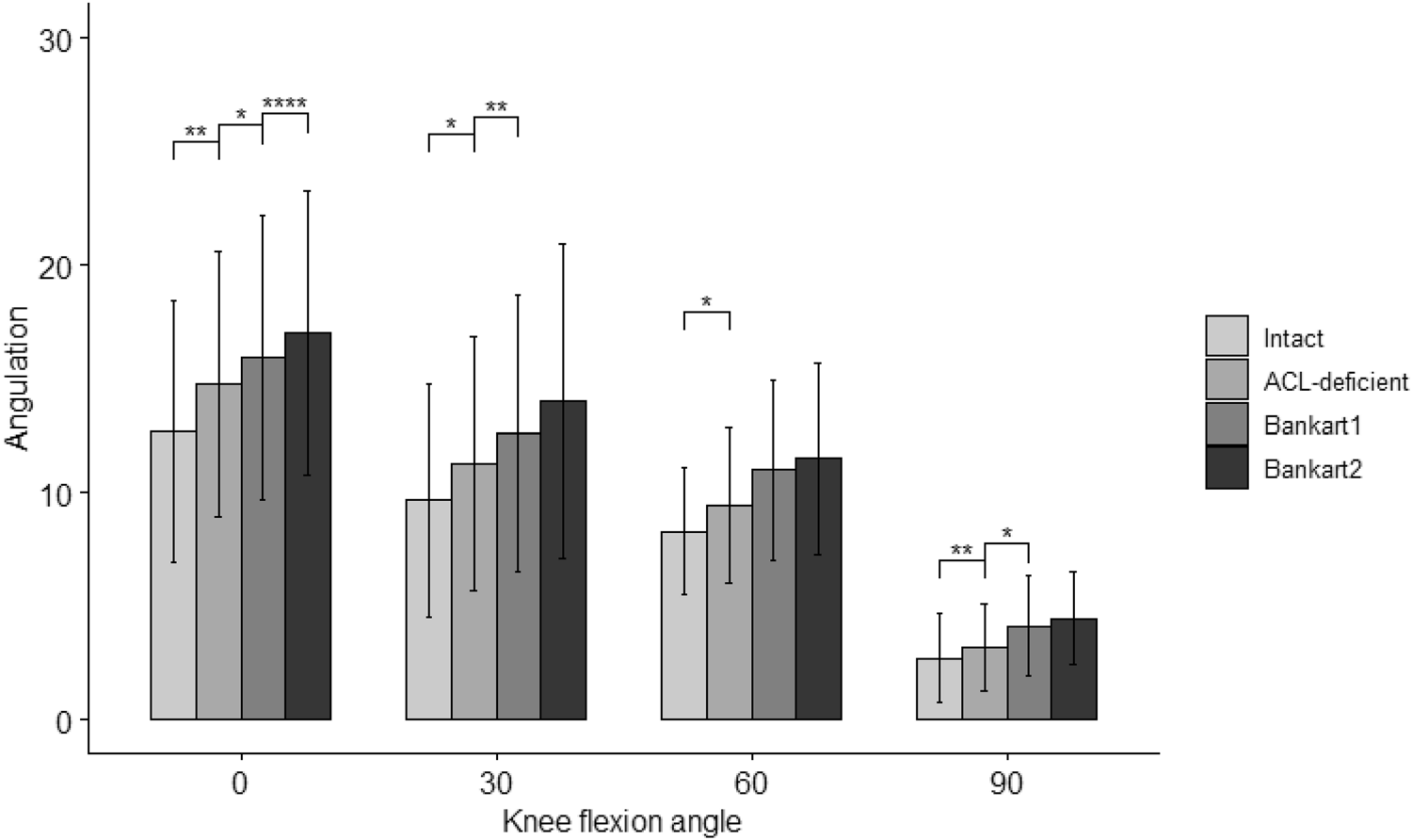
Internal rotation of the tibia during a 4 Nm internal rotational torque in Intact, ACL-deficient, Bankart 1 and 2 states in 0°–90° of knee flexion. Statistically significant differences compared to the previous states are indicated (*p < 0.05, **p < 0.01, ***p < 0.001). Error bars indicate the standard deviation

Regarding the internal rotation of the tibia, there was a statistically significant interaction between effect of cutting state and flexion angle on the rotation of the tibia (*p* = 0.0002, eta2[g] = 0.01). Pairwise comparisons showed that the mean IR of the tibia was significantly different between all states at 0°, with the highest significance between the Bankart 1 and Bankart 2 states, meaning that both grades of PLTP fractures led to a statistically significant increase in internal angulation of the tibia. At 30° and 90° of knee flexion, the Bankart 1 impression fracture yielded a further increase in internal angulation, as shown in Fig. 5 and Table 2.

### Pivot-shift test: 4 Nm tibial IR and 8 Nm valgus torque

#### Anterolateral translation (ALT)

**Fig. 6:**
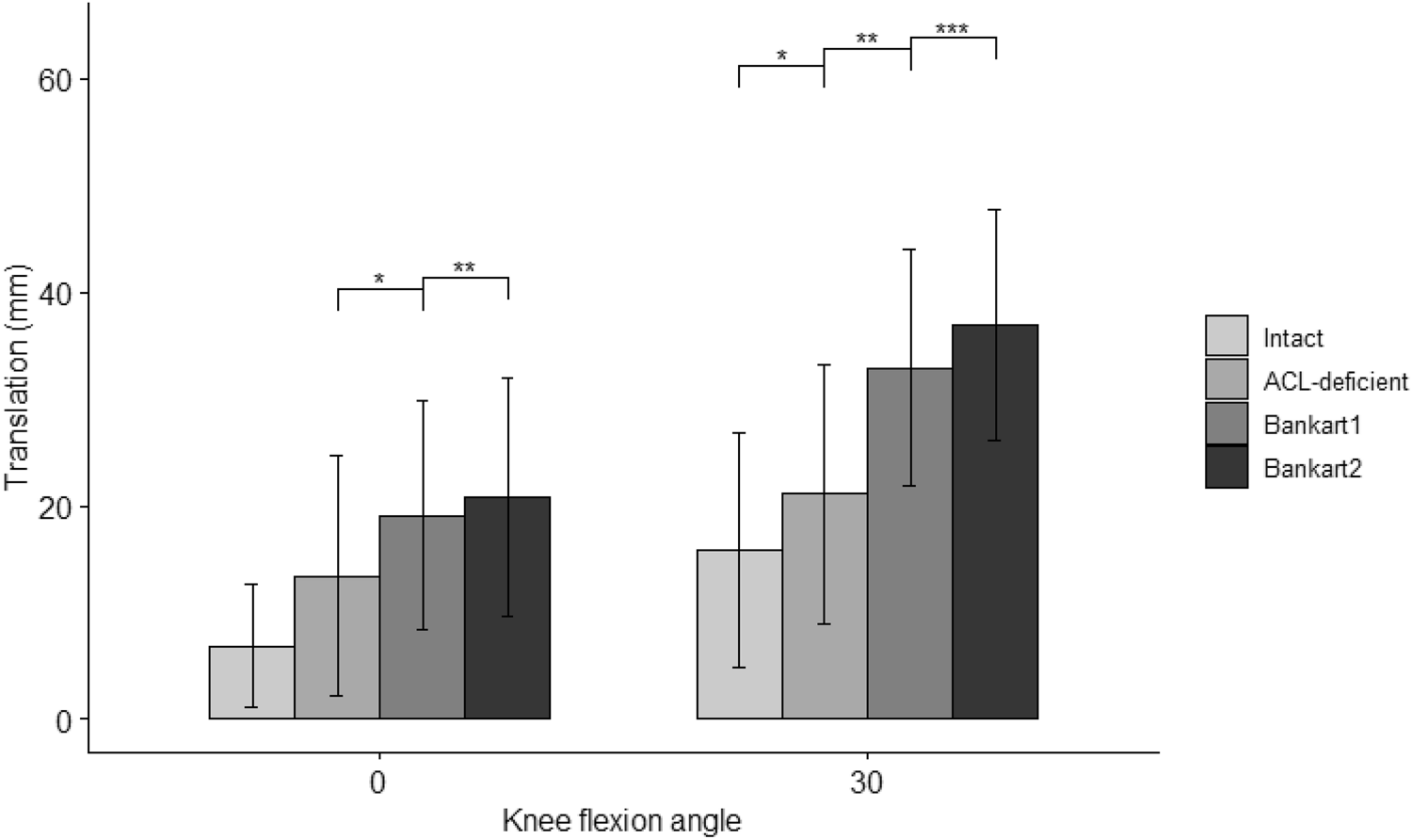
Anterolateral translation, i.e. translation of the midpoint of the lateral tibial plateau during a 4-Nm of tibial IR and 8-Nm valgus torque in 0° and 30° of knee flexion. Statistically significant differences compared to the previous states are indicated (*p < 0.05, **p < 0.01, ***p < 0.001). Error bars indicate the standard deviation

In terms of anterolateral translation, defined as the maximum translation of the midpoint of the lateral segment of the tibial plateau, there was no statistically significant interaction between effects of state and angle on translation. However, the effect of all cutting states was significant at fixed angles of both 0° and 30° (*p* = 0.002, eta2[g] = 0.26 for 0°, *p* = 4.31E−05 eta2[g] = 0.40 for 30°). Pairwise comparisons showed that the mean translation was significantly different between all states at 0° and 30° of flexion, except between the intact and ACL-deficient states at 0° (Fig. 6 and Table 3).

#### Anterolateral rotation (ALR)

**Fig. 7:**
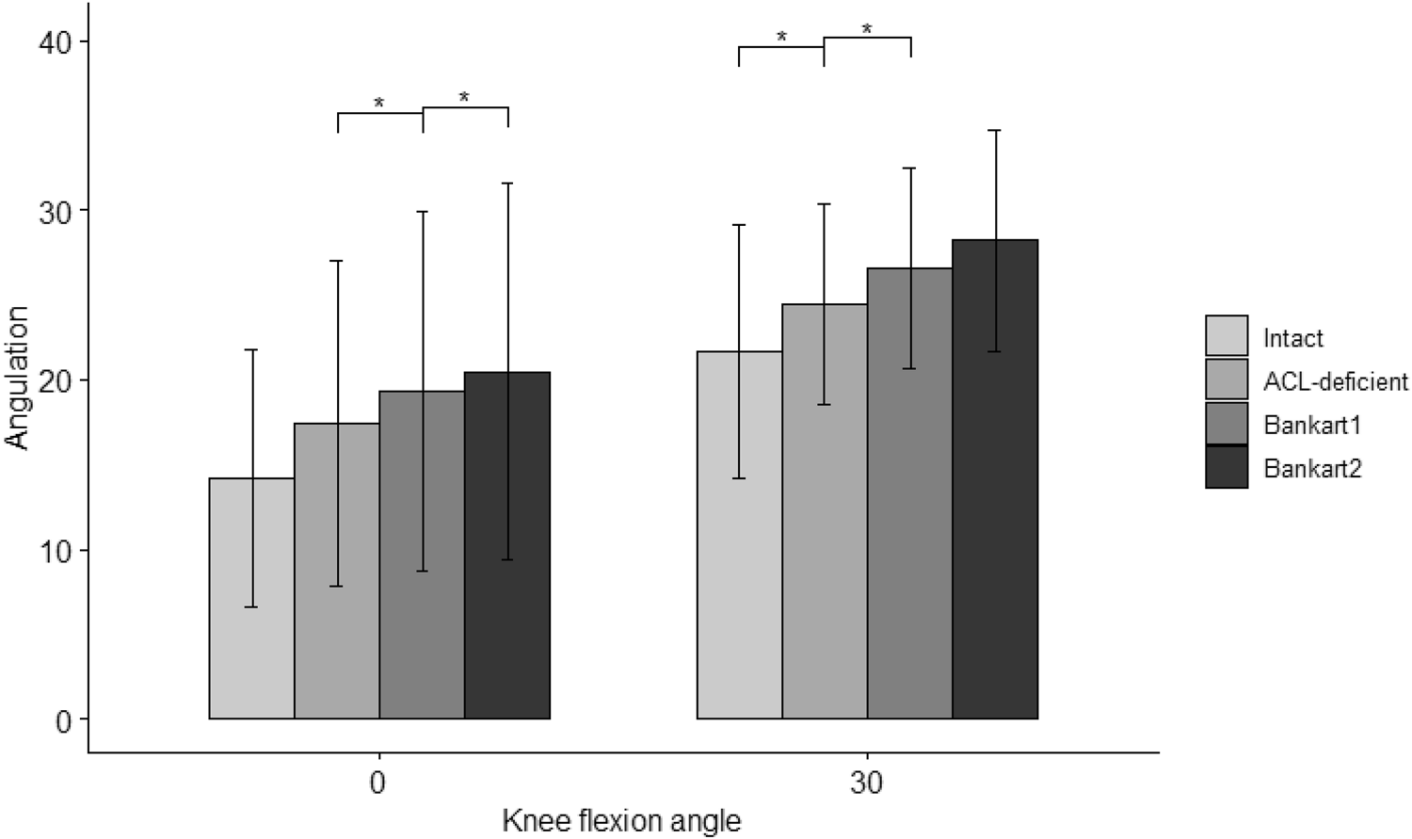
Anterolateral rotation, i.e. internal rotation of the tibia during a 4-Nm of tibial IR and 8-Nm valgus torque in 0° and 30° of knee flexion. Statistically significant differences compared to the previous states are indicated (*p < 0.05, **p < 0.01, ***p < 0.001). Error bars indicate the standard deviation

There was no statistically significant interaction effect of state and angle on anterolateral rotation, defined as the maximum rotation of the tibia around its longitudinal axis. However, the effect of state was significant at both fixed angles (*p* = 0.004, eta2[g] = 0.06 for 0°; *p* = 2.75E−07, eta2[g] = 0.14 for 30°). Pairwise comparisons showed that the mean angulation was significantly different between all states at angles of 0° and 30°, except between the intact and ACL-deficient states at 0° and between the Bankart 1 and Bankart 2 states at 30°, as shown in Fig. 7 and Table 4.

## Discussion

The results of this biomechanical study support the initial hypothesis that a high-grade PLTP fracture with consequent loss of bony support for the PHLM leads to an additional increase in ATT and ALR instability of the ACL-deficient joint.

Impression fractures of the PLTP, characterized by impression of the articular surface and impaction of the cortical bone of the tibial plateau, are among the wide variety of concomitant osteochondral lesions caused by the pivot-shift injury mechanism, one of the most intense injury types [3–6, 28, 33]. Nevertheless, in recent decades, studies investigating concomitant osteochondral injuries have focussed mostly on bone bruises, and very little attention has been given to impaction fractures of the tibial plateau [3–6, 19, 25, 28, 37, 39].

Smigielski et al. [9] presented at the ACL Study Group meeting in 2019 the first morphology-based classification based on their intraarticular depth in regards to the width of the PHLM. Accordingly, in grades I and II, there is still bony support of the PHLM left, whereas in grade III fractures, the osseous impression reaches up to the middle of (up to 50% of its width), and in the most severe cases (labelled grade IV), reaches or exceeds the full width of the PHLM (100% of its width), resulting in a complete loss of its osseous support (Fig. 1). Most recently, Bernholt et al. [4] investigated MRI scans of 825 patients with primary ACL ruptures and reported a high incidence of concomitant PLTP impression fractures (49.3%). Based on the morphological characteristics of the injuries, the authors proposed a classification system with 3 distinct categories and additional subcategories of the Type III fractures with a displaced bony fragment: Type IIIA with shear displaced fragments and Type IIIB with depressed fragments [4, 5].

However, to the best of our knowledge, no studies in the literature have investigated the biomechanical influence of these fractures on knee kinematics in the setting of ACL injury. To investigate their impact on the stability of the ACL-deficient joint, cadaveric knee specimens were subjected to simulated clinical tests from near extension to 90° of flexion, with a focus on Lachman, IR and pivot-shift tests. The pivot-shift test corresponding to a combined 4 Nm tibial IR and 8 Nm valgus torque was conducted at 0° and 30° of flexion to investigate the potential influence of these fractures on the ALR stability of the ACL-deprived joint by mimicking the dynamic multiplanar clinical pivot-shift test. Therefore, tibial movement was analyzed for anterolateral translation, defined as maximum translation of the midpoint of the lateral segment of the plateau, and ALR, defined as maximum rotation around the longitudinal axis of the tibia.

It has been reported that an increase in translation of the lateral compartment of the knee is directly correlated with the extent of knee pivoting, meaning that injuries to the lateral stabilizing structures of the knee, including the anterolateral capsule with the anterolateral capsular ligament (ALCL), ITT and LM [1, 2, 17, 23, 24], as well as increase in tibial slope result in higher grades of ALR instability [12, 13, 26].

Regarding the results of this study, it is important to emphasize the important role of LM in controlling the stability of the knee joint. In addition to its primary biomechanical role in shock absorption, load-bearing and load distribution, LM has been shown to have a significant role in controlling the ALR stability [8, 17, 27, 34]. However, the stability of the LM is crucial for maintaining its biomechanical function. This is attained through bony support by the lateral tibial plateau, integrity of the meniscofemoral ligaments (MFL) and anchoring of its roots [10, 14, 18, 24]. In terms of the correlation between injuries of the LM and PLTP impression fractures, Bernholt et al. [4] found an increased incidence of LM tears in patients with depressed fragment fractures (IIIB), as well as an increased incidence of lateral meniscus posterior horn and root lesions in patients with shear fragment fractures (IIIA). Furthermore, in a separate study evaluating the clinical effects of these fractures as concomitant injuries of an ACL rupture, Bernholt et al. [3] found that knees with high-grade impaction fractures had an increased likelihood of high-grade pivot-shift measurements preoperatively. Accordingly, in the current study, the biomechanical influence of depression fractures that mimicked the Type III fractures of the aforementioned classification were analyzed under the assumption that this degree of depression would lead to significant loss of osseous support of the LM and consequently alter its biomechanical function. The results of the present study demonstrated this clinical correlation in terms of the effects of high-grade fractures on the ALR instability of ACL-deprived knees from a biomechanical standpoint.

As with the shoulder joint, where the intact osseous support of the bony glenoid helps maintain the biomechanical function of the labrum, a posterolateral tibia plateau impression fracture seems to have the same effect as a bony Bankart lesion [21, 32, 35], resulting in instability of the joint by destabilizing the PHLM; this analogy served as the origin for the derived term “Bankart knee”.

Regarding the potential effects of these fractures on the clinical outcomes following isolated ACL-R, Bernholt et al. [3] found that high-grade PLTP fractures are associated with lower Lysholm scores at 2 years postoperatively, suggesting that these fractures could have significant clinical relevance. This was further confirmed in the recent study from Flury et al. [13], who showed that the extent of these fractures correlates with the injury to the anterolateral complex and has an impact on functional outcomes following an ACL reconstruction. However, additional clinical studies are necessary to conclusively determine whether an isolated ACL-R in cases of high-grade PLTP fracture concomitancy can fully restore the stability of the joint and, if not, what effect this potential residual laxity has on the postoperative functional and patient-reported outcomes.

There are several limitations of this study that must be taken into account: (1) A limited number of knees (*n* = 8) were tested due to the limited availability of cadaver specimens in our institution. (2) The mean age of the tested specimens was 77.5 years of age, which is significantly older than the characteristic age range when these injuries occur in the younger active patient population. Even though the bone quality of specimens was radiographically assessed, the greater age of the cadaver knees raises questions regarding whether possible overall lower bone quality led to further plateau depression during testing. (3) The tests were conducted under in vitro conditions. (4) The simulated tests were conducted in a static setup; thus, the reported values could differ from soft tissue navigation that allows for the analysis of kinematics during dynamic tests. (5) The standard proportions of the depression (15 mm width/10 mm height) could be considered exaggerated and not completely representative of the dimensions of the fractures that are most commonly seen in everyday practice. In this regard, Bernholt et al. [4] found that Type IIIA displaced fractures had the lowest incidence, meaning that fractures of this grade will not regularly be encountered. However, the goal of this study was to investigate the biomechanical influence of impaction fractures in a worst-case scenario, and the proportions of depressions were based on an analysis of MRIs from several patients treated in our institution for acute ACL rupture. (6) The axial load of 200 N was based on the loading capabilities of the testing robot along the longitudinal axis. Although this load is not a complete representation of the physiological weight-bearing conditions of the knee, it is useful for understanding the relative effects of axial loading on tibial translation and rotation.

## Conclusion

This study demonstrates that high-grade impression fractures of the posterolateral aspect of the tibial plateau increase the instability of ACL-deficient knees and result in an increase in translational and anterolateral rotational instability.

### Electronic supplementary material

Below is the link to the electronic supplementary material.

Supplementary file1 (DOCX 157 KB)

## Data Availability

Raw data can be made available upon request.

## Appendix I

Summary of pairwise comparisons analysis using paired *t* test between (see Tables 1, 2, 3 and 4).

**Table 1:** Lachman test: anterior tibial translation (89N)

Angle°	State	State	*p* value
0	Intact	ACL-deficient	0.008
0	ACL-deficient	Bankart1	0.007
0	Bankart1	Bankart2	0.000587
30	Intact	ACL-deficient	0.029
30	ACL-deficient	Bankart1	0.02
30	Bankart1	Bankart2	0.049
60	Intact	ACL-deficient	n.s
60	ACL-deficient	Bankart1	0.004
60	Bankart1	Bankart2	n.s
90	Intact	ACL-deficient	0.024
90	ACL-deficient	Bankart1	n.s
90	Bankart1	Bankart2	0.023

**Table 2:** Internal rotation (IR): 4 Nm of rotational torque

Angle°	State	State	*p* value
0	Intact	ACL-deficient	0.001
0	ACL-deficient	Bankart1	0.018
0	Bankart1	Bankart2	3.07e-05
30	Intact	ACL-deficient	0.015
30	ACL-deficient	Bankart1	0.009
30	Bankart1	Bankart2	n.s
60	Intact	ACL-deficient	0.035
60	ACL-deficient	Bankart1	n.s
60	Bankart1	Bankart2	n.s
90	Intact	ACL-deficient	0.005
90	ACL-deficient	Bankart1	n.s
90	Bankart1	Bankart2	n.s

**Table 3:** Anterolateral translation (ALT)

Angle°	State	State	*p* value
0	Intact	ACL-deficient	n.s
0	ACL-deficient	Bankart1	0.016
0	Bankart1	Bankart2	0.007
30	Intact	ACL-deficient	0.011
30	ACL-deficient	Bankart1	0.006
30	Bankart1	Bankart2	0.000374

**Table 4:** Anterolateral rotation (ALR)

Angle°	State	State	*p* value
0	Intact	ACL-deficient	n.s
0	ACL-deficient	Bankart1	0.022
0	Bankart1	Bankart2	0.023
30	Intact	ACL-deficient	0.019
30	ACL-deficient	Bankart1	0.01
30	Bankart1	Bankart2	n.s

## Declarations

### Award Paper

The study has been awarded with the “Albert Trillat Young Investigator's Award” at the 13th Biennial ISAKOS Congress, and the “1st place Research award” at the 38. AGA (Gesellschaft für Arthroskopie und Gelenkchirurgie) Congress in Innsbruck, Austria.

### Proof-reading

The manuscript was edited for proper English language, grammar, punctuation, spelling, and overall style by one or more of the highly qualified native English speaking editors at SpringerNature Author services proof-reading tool (see Supplement file).
