# Supplementary material for: The “Bankart knee”: high-grade impression fractures of the posterolateral tibial plateau lead to increased translational and anterolateral rotational instability of the ACL-deficient knee
Source: Knee Surg Sports Traumatol Arthrosc. 2023 May 8;31(10):4151–61. doi: 10.1007/s00167-023-07432-w (PMC10471664; doi:10.1007/s00167-023-07432-w)
Supplement: Supplementary file 1 — Supplementary file1 (DOCX 157 KB) [file 167_2023_7432_MOESM1_ESM.docx]

**Statistical analysis supplement file**

**Computer software summary**

This analysis was performed using R Core Team ^1^software, with additional packages tidyverse^2^, ggpubr^3^, rstatix^4^, readxl^5^.

1. R Core Team (2020). R: A language and environment for statistical computing. R Foundation for Statistical Computing, Vienna, Austria. URL <https://www.R-project.org/>.
2. Wickham et al., (2019). Welcome to the tidyverse. Journal of Open Source Software, 4(43), 1686, <https://doi.org/10.21105/joss.01686>
3. Alboukadel Kassambara (2020). ggpubr: 'ggplot2' Based Publication Ready Plots. R package version 0.4.0. <https://CRAN.R-project.org/package=ggpubr>
4. Alboukadel Kassambara (2020). rstatix: Pipe-Friendly Framework for Basic Statistical Tests. R package version 0.6.0. <https://CRAN.R-project.org/package=rstatix>
5. Hadley Wickham and Jennifer Bryan (2019). readxl: Read Excel Files. R package version 1.3.1. <https://CRAN.R-project.org/package=readxl>

# **Statistical analysis summary**

Prior to performing the statistical tests, the data was checked for normality (using the Shapiro-Wilk normality test and by visual inspection using QQ plot) and extreme outliers were removed (based on the boxplot method, i.e. values above Q3 + 3xIQR or below Q1 - 3xIQR).

A two-way repeated measures ANOVA with post-hoc Bonferroni corrections for multiple comparisons was performed to evaluate the effect of different Cutting states over different knee flexion angles on tibial translation/rotation. The two independent factors were the 4 states (intact, ACL-deficient, Bankart 1 and 2) and the knee angle (0°,30°,60° and 90°) within the same specimen, while the dependent variable was the resulting translation/rotation.

In the cases where the two-way repeated measures ANOVA did not detect a statistically significant interaction between the Cutting state and  flexion angle on translation or rotation of the lateral tibial, one-way repeated measures ANOVA was performed to evaluate the effect of different flexion angles or different cutting states on the translation/rotation separately.

One-tailed paired Student t-tests were used compare the mean translation/rotation between all of the fixed state/angle combinations.

The significance level was set to 0.05.

A post-hoc power analysis was performed using G*Power according to the study by Faul F et al. using the results of the simulated pivot shift test at 0° and 30° of flexion. Based on the reported data of this study, an effect size of 0.5 and 0.8 was obtained. With this effect sizes and an alpha of 0.05 the achieved power was 0.85 and 0.99, respectively.

# **Lachman test - Anterior tibial translation (89N)**

There was a statistically significant interaction between state and angle on translation, p < 0.05 (*Table 1)*. Therefore, the effect of state variable was analyzed at each angle. P-values were adjusted using the Bonferroni multiple testing correction method. The effect of state was significant at every fixed angle (eta2[g]=0.34 for 0°, eta2[g]=0.35 for 30°, eta2[g]=0.39 for 60°, eta2[g]=0.54 for 90°) (*Table 2*).

*Table 1 Table 2*

| **Effect** | **p-values** |
| --- | --- |
| state | 8.07E-08 |
| angle | 0.001 |
| state:angle | 0.012 |

| **Angle (°)** | **Effect** | **p- value** |
| --- | --- | --- |
| 0° | Cutting state | 0.000879 |
| 30° | Cutting state | 0.003 |
| 60° | Cutting state | 0.000965 |
| 90° | Cutting state | 0.000522 |

Pairwise comparisons, using paired t-test, show that the mean translation was significantly different between all states at the angles of 0° and 30°, between ACL-deficient and Bankart 1 at 60°, as well as between Intact – ACL-deficient and Bank1- Bankart 2 at 90° (*Figure 1*).

**Anterior tibial translation (89N)**


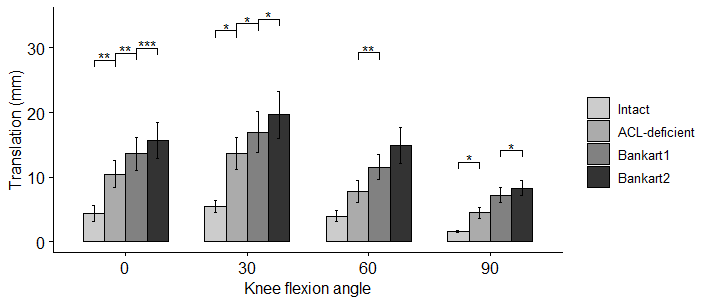


*Figure 1 Anterior translation of the tibia in Intact, ACL-deficient, Bankart 1 and 2 States at 0 to 90° of knee flexion. Statistically significant differences compared to the previous states are indicated (*p < 0.05, **p < 0.01, ***p < 0.001). Error bars indicate the standard error of the mean.*

One-way ANOVA of the different angles of flexion (0° to 90°) on each fixed state shows that there is a statistically significant effect of the flexion angle at each Cutting state (*Table 3*). The paired t-tests detect statistically significant difference of the mean tibial translation 30° and 90° for every state, as well as between 30° and 60° for ACL-deficient state (*Figure 2*).

*Table* 3

| **Cutting state** | **Effect** | ***P-value*** |
| --- | --- | --- |
| **Intact** | Angle (°) | 0.004 |
| **ACL-deficient** | Angle (°) | 0.000916 |
| **Bankart1** | Angle (°) | 0.004 |
| **Bankart2** | Angle (°) | 0.005 |

**Anterior tibial translation (89N)**


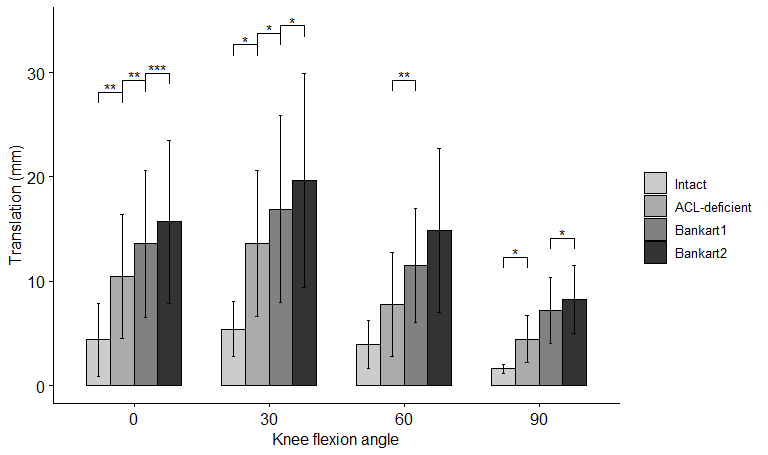


# *Figure 2 Anterior translation of the tibia during a simulated Lachman test at 0 to 90° of knee flexion in each of the cutting states. Statistically significant differences compared to the previous states are indicated (*p < 0.05, **p < 0.01, ***p < 0.001). Error bars indicate the standard error of the mean*

# **Posterior Drawer Test**

# **Posterior tibial translation (89N)**

There was NO statistically significant interaction between state and angle on translation (*Table* 4). Furthermore, the effect of state was NOT significant at any fixed angle (*Table 5*).

| **Angle** | **Effect** | **p** |
| --- | --- | --- |
| 0° | state | 0.364 |
| 30° | state | 0.353 |
| 60° | state | 0.333 |
| 90° | state | 0.886 |

*Table 4 Table 5*

| **Effect** | **p** |
| --- | --- |
| Cutting state | 0.243 |
| Angle (°) | 0.052 |
| Cutting State:Angle | 0.503 |

Pairwise tests did NOT show any difference between the mean translation for different states at any fixed angle (*Figure 3*).

**Posterior tibial translation (89N)**


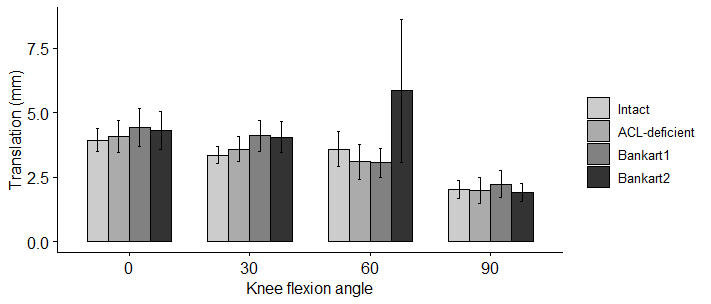


*Figure 3 Posterior translation of the tibia during a simulated Posterior Drawer test in Intact, ACL-deficient, Bankart 1 and 2 States at 0 to 90° of knee flexion. Statistically significant differences compared to the previous states are indicated (*p < 0.05, **p < 0.01, ***p < 0.001). Error bars indicate the standard error of the mean.*

# **Internal rotation test**

# **IR of the Tibia- 4Nm of rotational torque**

There was a statistically significant interaction between state and angle of knee flexion on rotation of the tibia, p < 0.05, eta2[g] = 0.01 (*Table 6*). Therefore, the effect of state variable was analyzed at each angle. P-values were adjusted using the Bonferroni multiple testing correction method. The effect of state was significant at every fixed angle of flexion (eta2[g]=0.08 for 0°, eta2[g]=0.08 for 30°, eta2[g]=0.12 for 60°, eta2[g]=0.12 for 90°) (*Table 7*).

*Table 6 Table 7*

| **Effect** | **p-values** |
| --- | --- |
| Cutting state | 3.78E-09 |
| Angle (°) | 3.90E-05 |
| state:angle | 0.000152 |

| **Angle (°)** | **Effect** | **p-values** |
| --- | --- | --- |
| 0° | Cutting state | 7.96E-06 |
| 30° | Cutting state | 0.000381 |
| 60° | Cutting state | 0.000852 |
| 90° | Cutting state | 0.003 |

Pairwise comparisons, using paired t-test, show that the mean rotation was significantly different between the following states (*Figure 4*):

**Internal rotation- 4 Nm of rotational torque**


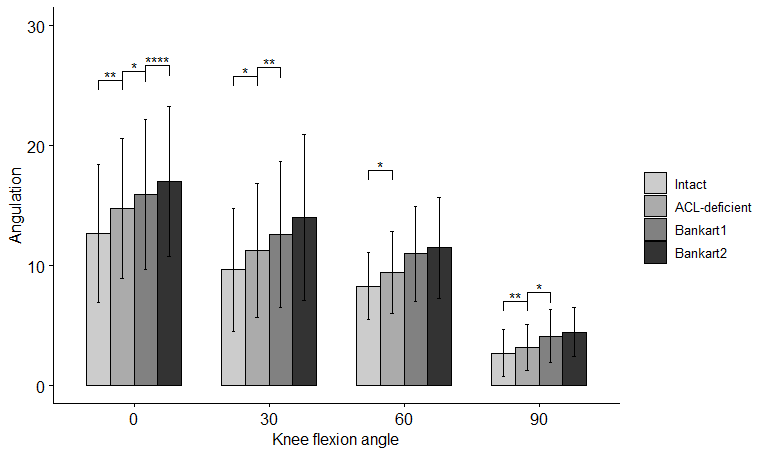


*Figure 4 Internal rotation (Angulation) of the tibia during a 4 Nm internal rotational torque in Intact, ACL-deficient, Bankart 1 and 2 states in 0° to 90° of knee flexion.. Statistically significant differences compared to the previous states are indicated (*p < 0.05, **p < 0.01, ***p < 0.001). Error bars indicate the standard error of the mean.*

The effect of the angle on the angulation is significant in all of the states (*Table 8*). The paired t-tests detect statistically significant difference of the mean rotation of the tibia in the knee flexion degrees shown on the *Figure 5*.

*Table 8*

| **Cutting state** | **Effect** | **p-values** |
| --- | --- | --- |
| **Intact** | Angle (°) | 0.000177 |
| **ACL-deficient** | Angle (°) | 3.31E-05 |
| **Bankart1** | Angle (°) | 3.60E-05 |
| **Bankart2** | Angle (°) | 2.44E-05 |

**Internal rotation- IR 4Nm of rotational torque**


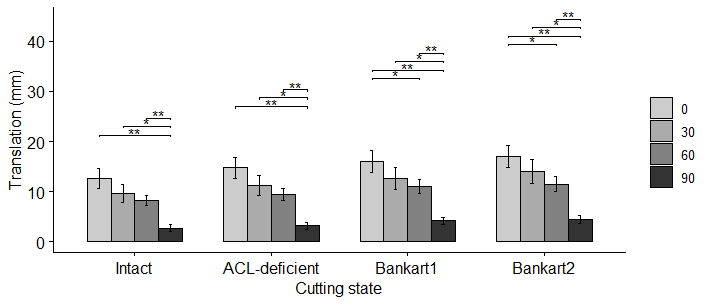


*Figure 5 Internal rotation (Angulation) of the tibia during a 4 Nm internal rotational torque in 0 to 90° of knee flexion Statistically significant differences compared to the previous states are indicated (*p < 0.05, **p < 0.01, ***p < 0.001). Error bars indicate the standard error of the mean*.

# **External rotation**

# **ER of the Tibia- 4 Nm of rotational torque**

There was NO statistically significant interaction between state and angle of knee flexion on rotation of the tibia (*Table 9)*. Furthermore, the effect of state was NOT significant at any fixed angle, except at 0° (*Table 10*).

*Table 9 Table 10*

| **Effect** | **p-values** |
| --- | --- |
| State | 0.002 |
| Angle (°) | 0.003 |
| State:Angle | 0.537 |

| **Angle (°)** | **Effect** | **p-values** |
| --- | --- | --- |
| 0 | Cutting state | 0.037 |
| 30 | Cutting state | 0.072 |
| 60 | Cutting state | 0.494 |
| 90 | Cutting state | 0.46 |

Pairwise tests did NOT show any difference between the mean translation for different states at any fixed angle, except between Bankart 1 and Bankart 2 at 30° (*Figure 8*).

**External rotation- 4 Nm of rotational torque**
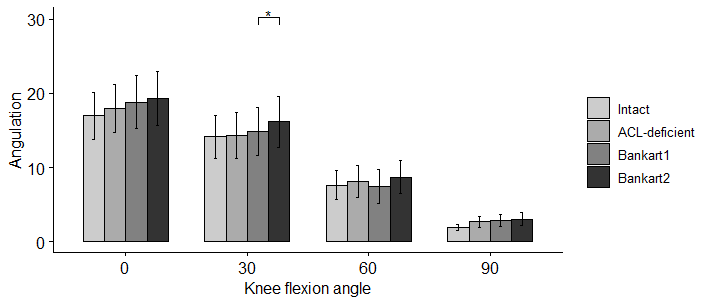


*Figure 6 External rotation (Angulation) of the tibia during a 4 Nm external rotational torque (IR) in Intact, ACL-deficient, Bankart 1 and 2 states in 0° to 90° of knee flexion.. Statistically significant differences compared to the previous states are indicated (*p < 0.05, **p < 0.01, ***p < 0.001). Error bars indicate the standard error of the mean*

# **Simulated Pivot-shift Test**

# **Anterolateral translation (translation of the midpoint of LTP) during a combined 4 Nm of tibial IR and 8Nm valgus torque**

There was NO statistically significant interaction between state and angle on the translation of the midpoint of the lateral tibial plateau (*Table 11*). However, the effect of state was significant at both fixed angles (eta2[g]=0.26 for 0°, eta2[g]=0.40 for 30°) (*Table 12*).

*Table 11 Table 12*

| **Effect** | **p-values** |
| --- | --- |
| Cutting state | 1.76e-06 |
| Angle (°) | 0.007 |
| State: Angle | 0.06 |

| **Angle (°)** | **Effect** | **p-values** |
| --- | --- | --- |
| 0 | Cutting state | 0.002 |
| 30 | Cutting state | 4.31E-05 |

Pairwise comparisons, using paired t-test, show that the mean translation was significantly different between all states at the angles of 0° and 30°, except between Intact and ACL-deficient at 0° *(Figure 7*).

**Pivot-shift Test (4 Nm of tibial IR and 8Nm valgus torque)**


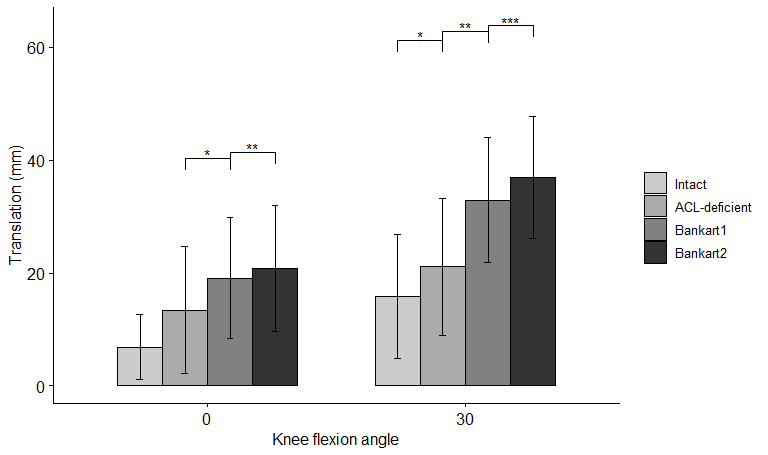


*Figure 7 Anterolateral translation, i.e. translation of the midpoint of the lateral tibial plateau during a 4 Nm of tibial IR and 8 Nm valgus torque in 0° and 30° of knee flexion. Statistically significant differences compared to the previous states are indicated (*p < 0.05, **p < 0.01, ***p < 0.001). Error bars indicate the standard error of the mean.*

The effect of the angle on the ALT is significant for Bankart 1 and Bankart 2 (*Table 13*). The paired t-tests confirm this (*Figure 8*):

*Table 13*

| **State** | **Effect** | **p-values** |
| --- | --- | --- |
| **Intact** | Angle | 0.067 |
| **ACL-deficient** | Angle | 0.097 |
| **Bankart1** | Angle | 0.002 |
| **Bankart2** | Angle | 0.000677 |

**Pivot-shift Test (4Nm of tibial IR and 8-Nm valgus torque)**

**
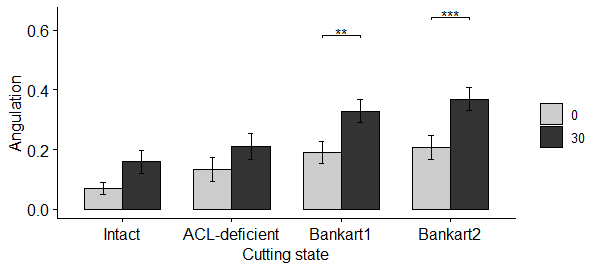
**

*Figure 8 Anterolateral translation, i.e. translation of the midpoint of the lateral tibial plateau during a combined 4 Nm of tibial IR and 8 Nm valgus torque in 0° and 30° of knee flexion. Statistically significant differences compared to the previous states are indicated (*p < 0.05, **p < 0.01, ***p < 0.001). Error bars indicate the standard error of the mean.*

# **Simulated Pivot-shift Test**

**Anterolateral rotation (IR of the tibia) during a combined 4Nm of tibial IR and 8-Nm valgus torque**

There was NO statistically significant interaction between state and angle on rotation of the tibia (*Table* *14*). However, the effect of state was significant at both fixed angles (eta2[g]=0.06 for 0°, eta2[g]=0.14 for 30°) (*Table* *15*).

*Table 14*  *Table 15*

| **Effect** | **p-values** |
| --- | --- |
| State | 5.63e-09 |
| Angle | 0.02 |
| state:angle | 0.79 |

| **Angle** | **Effect** | **p-values** |
| --- | --- | --- |
| 0 | State | 0.004 |
| 30 | State | 2.75E-07 |

Pairwise comparisons, using paired t-test, show that the mean angulation was significantly different between all states at the angles of 0° and 30°, except between Intact and ACL-deficient at 0° and Bankart1 and Bankart2 at 30° (*Figure 9*).

**Pivot-shift Test (4Nm of tibial IR and 8-Nm valgus torque)**


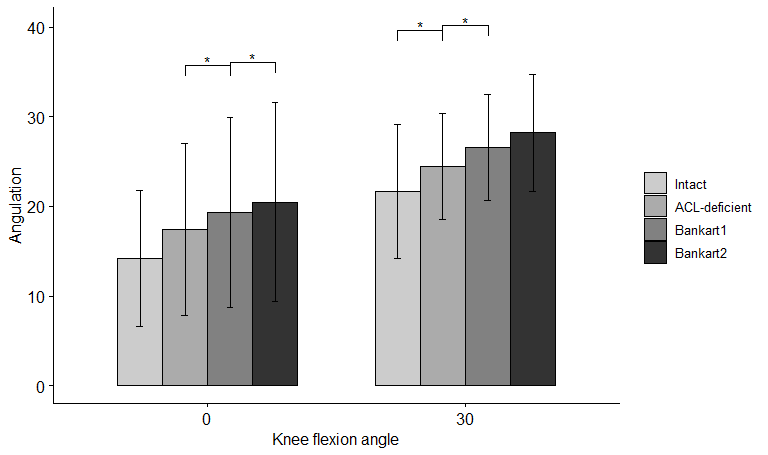


*Figure 9 Anterolateral rotation, i.e. internal rotation of the tibia during a 4-Nm of tibial IR and 8-Nm valgus torque in 0° and 30° of knee flexion. Statistically significant differences compared to the previous states are indicated (*p < 0.05, **p < 0.01, ***p < 0.001). Error bars indicate the standard error of the mean.*

The effect of the angle on the angulation is significant for all states (*Table 16*). The paired t-tests confirm this (*Figure 10*):

*Table 16*

| **State** | **Effect** | **p-values** |
| --- | --- | --- |
| **Intact** | angle | 0.016 |
| **ACL-deficient** | angle | 0.035 |
| **Bankart1** | angle | 0.036 |
| **Bankart2** | angle | 0.000677 |

**Pivot-shift Test (4Nm of tibial IR and 8-Nm valgus torque)**


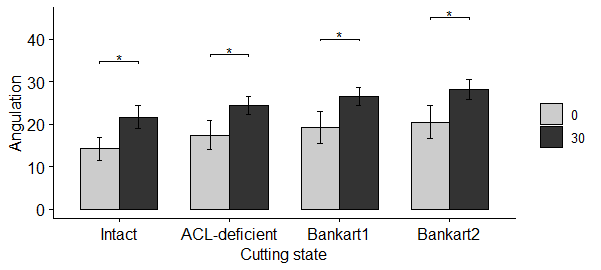


*Figure 10 Anterolateral rotation, i.e. internal rotation of the tibia during a combined 4-Nm of tibial IR and 8-Nm valgus torque in 0° and 30° of knee flexion. Statistically significant differences compared to the previous states are indicated (*p < 0.05, **p < 0.01, ***p < 0.001). Error bars indicate the standard error of the mean.*
